# Rhoptry kinase protein 39 (ROP39) is a novel factor that recruits host mitochondria to the parasitophorous vacuole of *Toxoplasma gondii*

**DOI:** 10.1242/bio.058988

**Published:** 2021-09-30

**Authors:** Junpei Fukumoto, Takaya Sakura, Ryuma Matsubara, Michiru Tahara, Motomichi Matsuzaki, Kisaburo Nagamune

**Affiliations:** 1 Department of Parasitology, National Institute of Infectious Diseases, Shinjuku-ku, Tokyo 162-8640, Japan; 2 Graduate School of Life and Environmental Sciences, University of Tsukuba, Tsukuba, Ibaraki 305-8572, Japan; 3 Faculty of Life and Environmental Sciences, University of Tsukuba, Tsukuba, Ibaraki 305-8572, Japan

**Keywords:** *Toxoplasma gondii*, Quantitative proteomics, ROP39, Host mitochondrial recruitment

## Abstract

Most intracellular pathogens replicate in a vacuole to avoid the defense system of the host. A few pathogens recruit host mitochondria around those vacuoles, but the molecules responsible for mitochondrial recruitment remain unidentified. It is only in the apicomplexan parasite *Toxoplasma gondii*, that mitochondrial association factor 1b (MAF1b) has been identified as an association factor for host mitochondria. Here, we show that rhoptry kinase family protein 39 (ROP39) induces host mitochondrial recruitment in *T. gondii*. We found that the abundance of ROP39 was increased on host mitochondria extracted from human foreskin fibroblasts (HFFs) infected with *T. gondii*. ROP39 expressed exogenously in HFFs localized on host mitochondria, indicating that it has the potential to bind to host mitochondria without assistance from other parasite factors. Confocal microscopy revealed that ROP39 colocalized with host mitochondria on the membrane of parasitophorous vacuoles, in which the parasites reside. Moreover, we observed about a 10% reduction in the level of mitochondrial association in *rop39*-knockout parasites compared with a parental strain.

## Abstract (precis)

**Summary:** We revealed that ROP39 recruitments host mitochondria, possibly through its kinase activity. Exploiting components involved with recruitment of host mitochondria advances the understanding of how the parasites employ mitochondrial recruitment to survive in host cells.

## INTRODUCTION

*Toxoplasma gondii* is an obligate intracellular protozoan parasite that can infect almost all warm-blooded animals, and nearly one-third of the world's population is infected with this parasite (Grigg and Sundar, 2009). *Toxoplasma gondii* causes a latent infection in most humans, but leads to lethal diseases, including encephalitis, in immunosuppressed people due to acquired immunodeficiency syndrome (AIDS) or organ transplantation. In pregnant women, initial infection with *T. gondii* may cause fetuses to encounter the parasites through vertical transmission, and this can result in serious symptoms such as retinochoroiditis, hydrocephalus and psychomotor retardation (Montoya and Liesenfeld, 2004).

*Toxoplasma gondii* is sequestered in host cells by the parasitophorous vacuole (PV), which permits intracellular replication of parasites. It is well known that *T. gondii* associates host mitochondria and endoplasmic reticulum (ER) around PVs (Sinai et al., 1997). Such mitochondrial association is also observed in other intracellular pathogens, such as *Legionella pneumophila*, *Chlamydia psittaci* and *Encephalitozoon cuniculi* (Horwitz et al., 1983; Matsumoto et al., 1991; Scanlon et al., 2004), whose molecular mechanism and function remains largely unclear. In a recent quantitative trait locus (QTL) analysis of *T. gondii*, mitochondrial association factor 1b (MAF1b) was identified in *T. gondii* as a parasite factor that associates host mitochondria around its PV membranes (PVMs) (Pernas et al., 2014). However, which factor(s) recruits host mitochondrial to PVMs remains unknow.

Rhoptry kinase family proteins (ROPs) are secretory proteins that are discharged into host cells from an apicomplexan parasite organelle called a rhoptry. Following the formation of PVMs, these molecules localize on PVMs or in PVs or host nuclei, and modify host function to optimize the milieu around parasites. ROP2 and ROP8 were suggested to induce mitochondrial association in an antisense-RNA based study (Sinai and Joiner, 2001), but this was not supported by use of a *rop2a/rop2b*/*rop8* triple knockout mutant (Pernas and Boothroyd, 2010). However, ROPs are still fascinating molecules with regard to their relevance in mitochondrial recruitment. ROPs are secreted into host cells immediately after invasion by *T. gondii* (Boothroyd and Dubremetz, 2008), and this corresponds to the observation that host mitochondrial association occurs within a few minutes after penetration of host cells (Sinai and Joiner, 2001). Thus, secretion of ROPs is coincident with onset of mitochondrial association. Moreover, *T. gondii* discharges many evacuoles containing ROPs into glycosylphosphatidylinositol-deficient cells, and the mitochondrial association is enhanced in these cells (Tahara et al., 2016). These observations may indicate a link between ROPs and mitochondrial recruitment. Considering MAF1b is a dense granule protein which is secreted after invasion, we speculated certain ROPs secreted into host cell prior to invasion may work to recruit host mitochondria to PVMs.

In this study, we search for ROPs whose abundance are upregulated on host mitochondria in *T. gondii* infection to find new factor(s) to induce recruitment of host mitochondria using a quantitative proteomics method and ROP39 was identified. As a result of the further analysis, we found ROP39 as a causative factor in host mitochondrial recruitment.

## RESULTS

### Increased abundance of ROP39 on host mitochondria during infection

To explore ROPs involved in host mitochondrial recruitment, we employed a quantitative proteomics method: isobaric tag for relative and absolute quantitation (iTRAQ) (Wiese et al., 2007) to identify ROPs with increased levels on host mitochondria. Host mitochondria were extracted from parasite-infected cells, and proteins were purified from them. The proteins were purified with the same way from a mixture of HFFs and parasites as a control to exclude non-specific binding. A total of 320 proteins of *T. gondii* were detected with an amino acid sequence of at least one peptide fragment that matched the reference sequence with >95% confidence. Among these proteins, eleven ROPs were found and only ROP39 (TGGT1_262050) was more abundantly detected from mitochondria isolated from *T. gondii*-infected cells (Table 1). We performed evacuole assays to check that ROP39 actually was injected into host cytosol prior to PVMs formation. We confirmed that cytochalasin treatment blocked the invasion of parasites but not the secretion of vacuole (evacuole), which indicates that ROP39 secretion occurs followed by the building up of PVMs (Fig. S1).

**Table 1.**
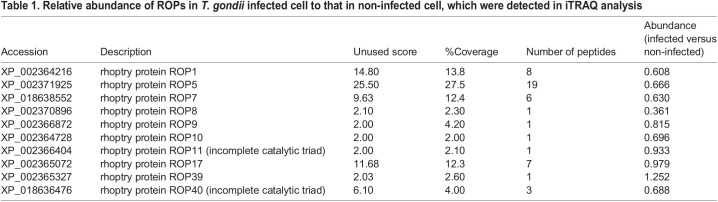
Relative abundance of ROPs in *T. gondii* infected cell to that in non-infected cell, which were detected in iTRAQ analysis

### ROP39 localizes on host mitochondria in mammalian cells

**Fig. 1.**
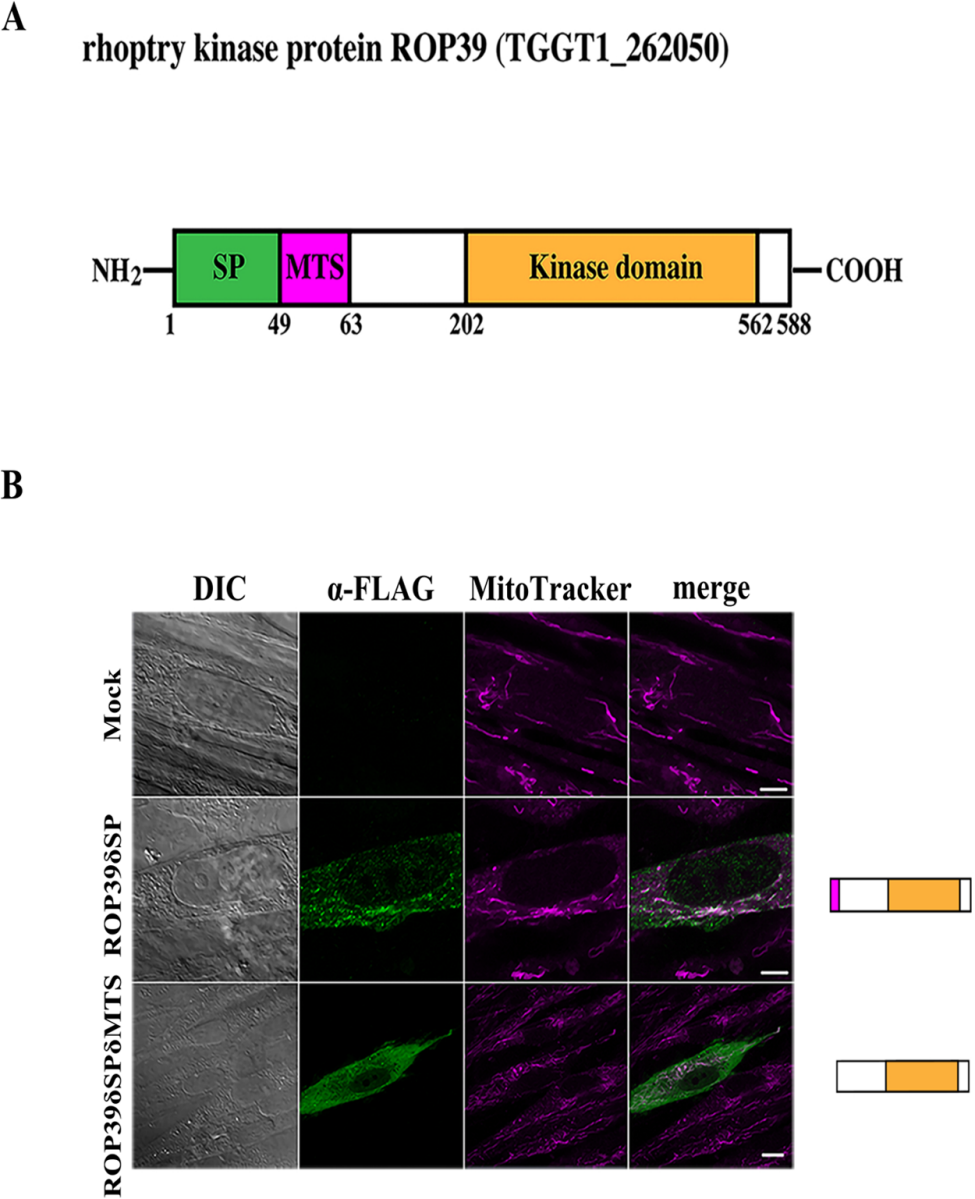
**ROP39 has the propensity to bind to host mitochondria.** (A) Schematic diagram of the domain structure of ROP39. The signal peptide (SP), mitochondria targeting signal (MTS), and kinase domain were predicted using SignalP 3.0 (http://www.cbs.dtu.dk/services/SignalP-3.0/), Mitoprot (https://ihg.gsf.de/ihg/mitoprot. html), and SMART (https://prosite.expasy.org/), respectively. (B) Representative immunofluorescence micrographs of HFFs transfected with mammalian expression vectors expressing FLAG-tagged ROP39δSP and ROP39δSPδMTS exogenously. Samples were stained for mitochondria with MitoTracker (magenta) and ROP39 with anti-FLAG antibody (green) and scanned using confocal microscopy. Scale bars: 2 μm.

To assess whether localization of ROP39 is due to its inherent properties or to the effects of other parasite-derived factors, we investigated the molecular structure of ROP39 to use a domain prediction tool. ROP39 has been predicted to have a signal peptide at amino acid positions 1-49 by SP-HMM/SP-NN (https://toxodb.org/toxo/). Moreover, the presence of a mitochondrial targeting sequence (MTS) in ROP39 was estimated on Mitoplot, a prediction tool to identify mitochondrial pre-sequence and cleavage sites (Fukasawa et al., 2015). A cleavage site in mitochondria was predicted for the peptide bond between amino acids 63 and 64 (Fig. 1A).

We expressed ROP39 lacking the signal peptide (ROP39δSP) exogenously in human foreskin fibroblasts (HFFs) using a mammalian expression vector. FLAG-tagged ROP39δSP was found to localize partially on host mitochondria (Fig. 1B). The pattern of partial colocalization of ROP39 and host mitochondria was similar to that of counter staining of ROP2 and mitochondria (Sinai and Joiner, 2001). Furthermore, we generated a vector to express ROP39 lacking the N-terminal 63 amino acids (ROP39δSPδMTS) based on the prediction of Mitoprot. The expressed protein ROP39δSPδMTS was scattered around the cytosol in HFFs (Fig. 1B). These findings suggest the possibility that ROP39 bind to host mitochondria in the cytosol through its MTS prior to the formation of PVMs.

### ROP39 colocalizes with host mitochondria on PVMs in *T. gondii*

**Fig. 2.**
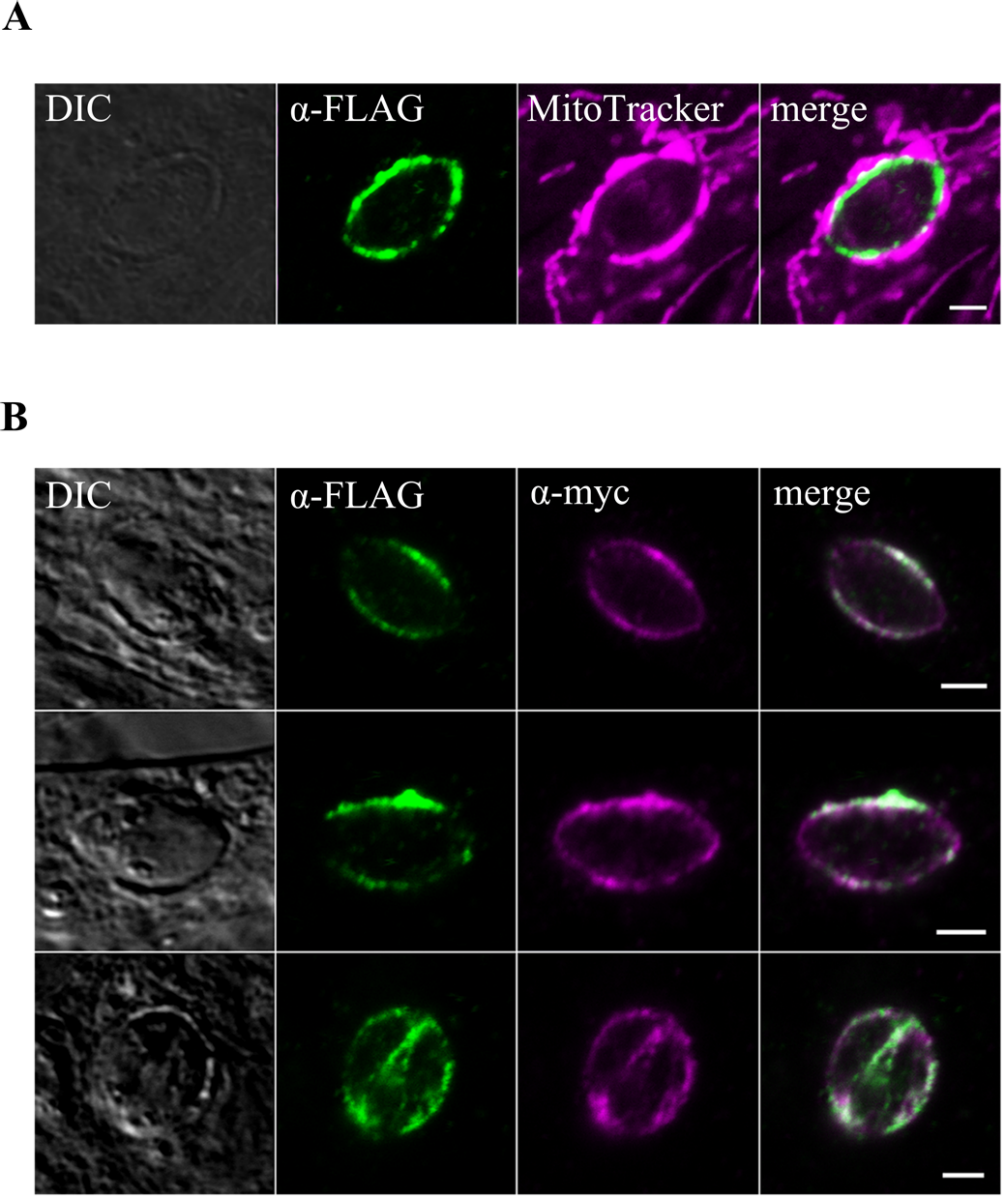
**ROP39 colocalizes partially with host mitochondria on PVMs.** (A) Representative immunofluorescence micrographs of human foreskin fibroblasts (HFFs) infected with *T. gondii* transiently expressing FLAG-tagged ROP39 under SAG1 promoter at 24 h post-infection. Samples were stained for mitochondria with MitoTracker (magenta) and ROP39 with anti-FLAG antibody (green) and scanned by confocal microscopy. Scale bar: 2 μm. (B) *T. gondii* transiently expressing FLAG-tagged ROP39 and myc-tagged MAF1b was counterstained with anti-FLAG antibody (green) and anti-myc antibody (magenta) at 24 h post-infection. Scale bars: 2 μm.

To investigate whether ROP39 is actually targeted to PVMs, we confirmed the localization of ROP39 in infected cells with *T. gondii*. ROP39 was found to be localized on PVMs and to partially colocalize with host mitochondria in cells (Fig. 2A). To further investigate the localization of ROP39, we labeled ROP39 with MAF1b (PVMs marker) by conducting an immunostaining (Fig. 2B). We observed that ROP39 colocalized with MAF1b on PVMs (Fig. 2B). These findings support the localization of ROP39 on PVMs and are consistent with the localization of ROP39 on host mitochondria indicated by the data from iTRAQ and Fig. 1.

### A *rop39*-knockout mutant exhibits decreased association of host mitochondria

**Fig. 3.**
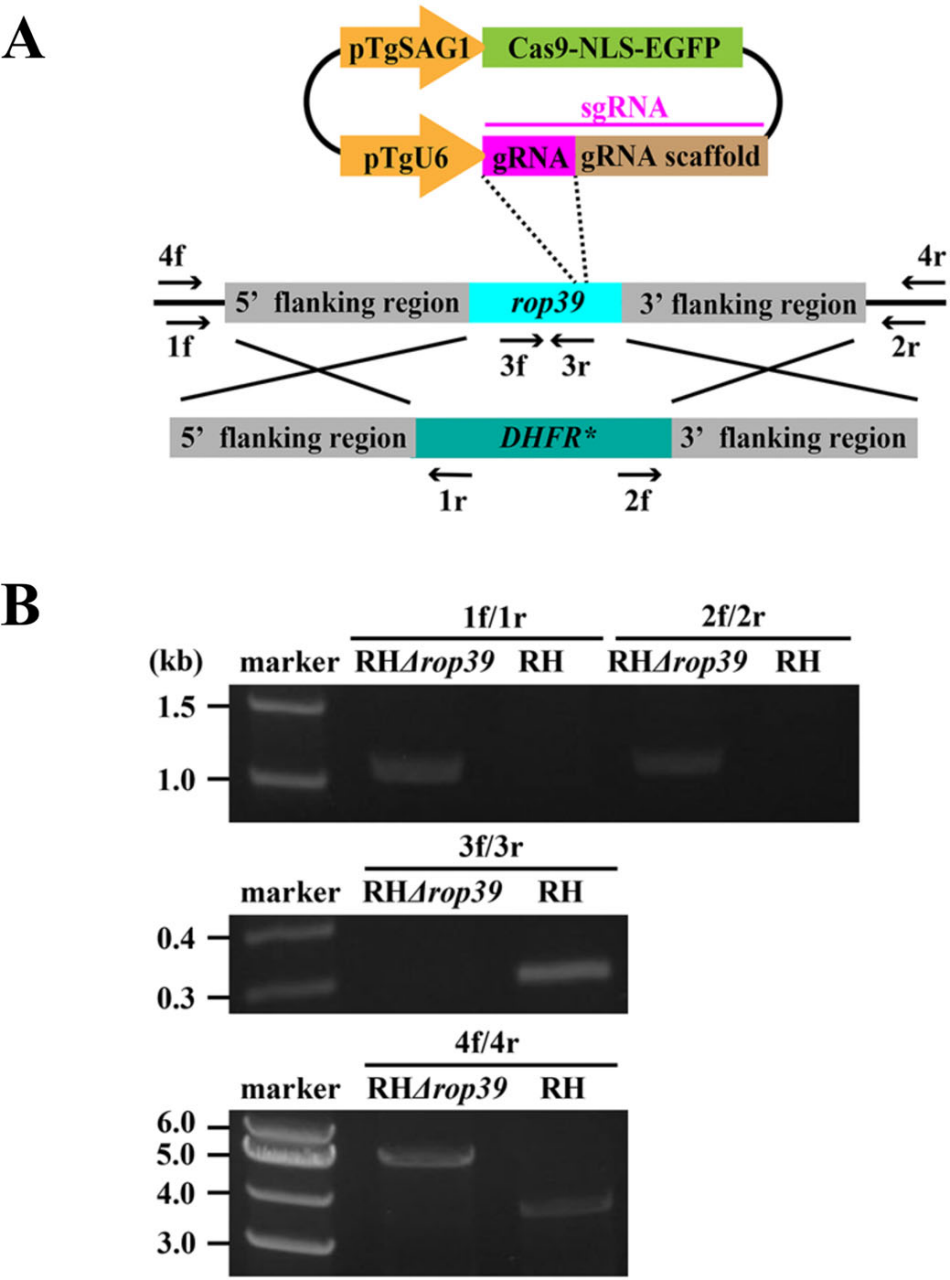
**Establishment of the *rop39*-deletion mutant RH*Δrop39* using CRISPR/Cas9.** (A) Schematic image of the gene knockout of *rop39* in the CRISPR/Cas9 system. Arrows indicate the primer-binding sites for PCR-based checking. (B) The *rop39* gene knockout was checked using PCR. DNA segments were amplified with 1f/1r (RH*Δrop39*, 1091 bp; RH, not amplified), 2f/2r (RH*Δrop39*, 1120 bp; RH, not amplified), 3f/3r (RH*Δrop39*, not amplified; RH, 294 bp), and 4f/4r (RH*Δrop39*, 5309 bp; RH, 3913 bp) primer sets.

**Fig. 4.**
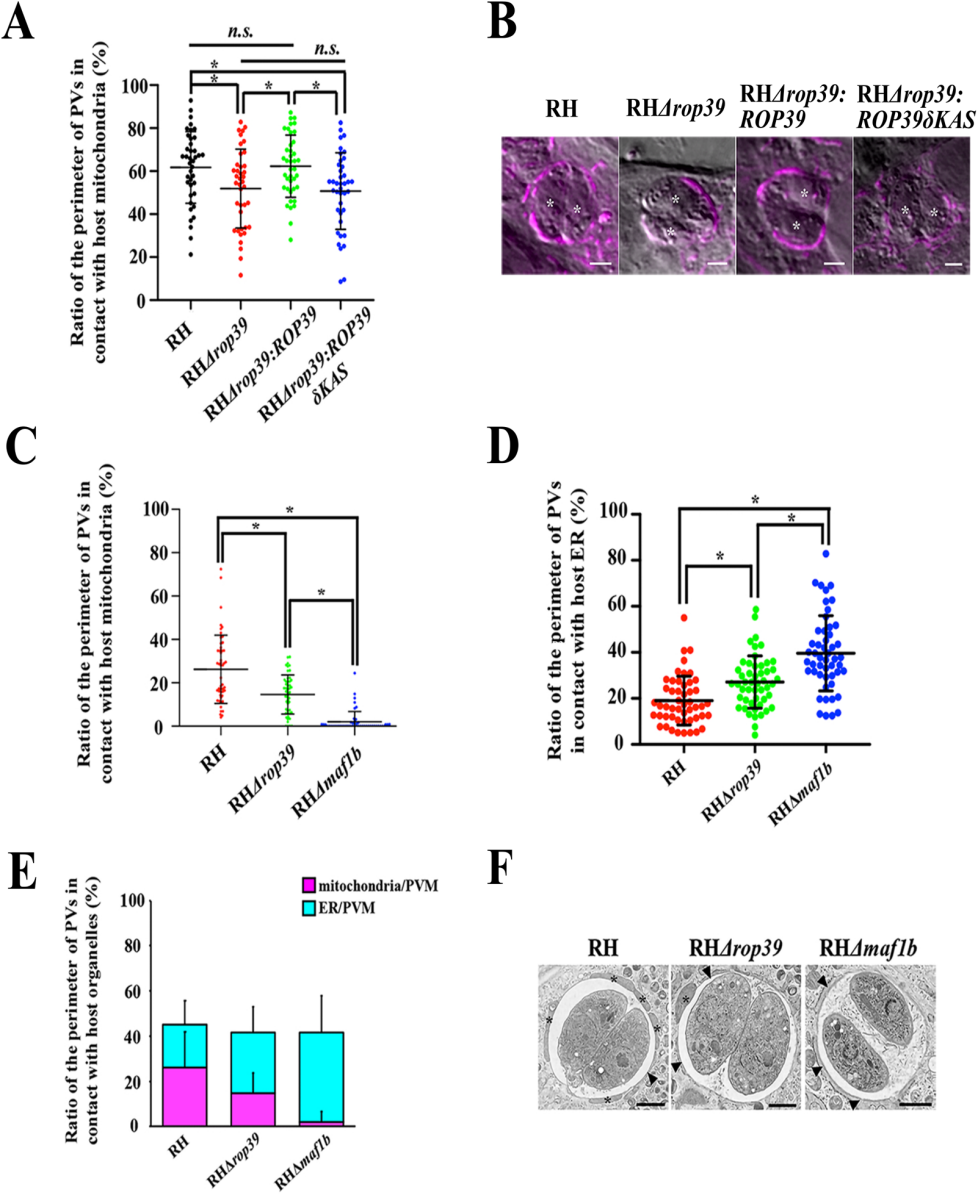
**Measurement of ratio of host mitochondria and ER in contact with PVs of *T. gondii* strains.** (A) Ratio of host mitochondria in contact with PVs of RH, RH*Δrop39*, RH*Δrop39:ROP39* and RH*Δrop39:ROP39δKAS* strains. Samples were scanned by confocal microscopy to obtain images for measurement. Each data point represents ratio of the length of host mitochondria in contact with PVs to the perimeter of PVs. The horizontal lines represent the mean±s.d. Combined data are from two independent experiments (*n*=40 per strain). Statistical significance was determined using one-way ANOVA with Tukey–Kramer post hoc test (*P<0.05). *n.s.*, not significant (*P*>0.05). (B) Representative confocal micrographs of host mitochondria in contact with PVs of RH, RH*Δrop39*, RH*Δrop39:ROP39* and RH*Δrop39δKAS* strains. Samples were stained for mitochondria with MitoTracker (magenta). Asterisks (*) indicate *T. gondii*. Scale bars: 2 μm. (C,D) Ratio of host mitochondria (C) or ER (D) in contact with PVs of RH, RH*Δrop39* and RH*Δmaf1b* strains. Samples were scanned by transmission electron microscope to obtain images for measurement. Each data point represents ratio of the length of host mitochondria or ER in contact with PVs to the perimeter of PVs. The horizontal lines represent the mean±s.d. (*n*=50 per strain). Statistical significance was determined using Mann–Whitney test (C, *P<0.001) or one-way ANOVA with Tukey–Kramer post hoc test (D, *P<0.01). (E) Ratio of host mitochondria and ER in contact with PVs of RH, RH*Δrop39* and RH*Δmaf1b* strains. The ratio was obtained by summing those of host mitochondria (B) and ER (C) of each strain. (F) Representative transmission electron micrographs of host mitochondria and ER in contact with PVs of RH, RH*Δrop39* and RH*Δmaf1b* strains. Asterisks (*) indicate host mitochondria and arrowheads point to host ER. Scale bars: 2 μm.

To show the relevance to ROP39 in the mitochondrial recruitment, we generated a *rop39* deficient mutant RH*Δrop39* in the RH strain background using a CRISPR/Cas9 system (Fig. 3A) and deletion of ROP39 was checked by PCR (Fig. 3B). We measured ratio of the perimeter of PVs in contact with host mitochondria and defined the ratio as the level of host mitochondrial association, which indicates the ability of ROP39 to recruit host mitochondria. Host mitochondrial association decreased by 10% in RH*Δrop39* (51.6±18.2%) compared with the parental RH strain (61.9±16.7%) in analysis of immunofluorescence microscopy images (*P*<0.05, one-way ANOVA followed by Tukey-Kramer post hoc test) (Fig. 3A). A RH*Δrop39* complemented strain, RH*Δrop39:ROP39*, showed complete rescue of mitochondrial association (62.2±14.5%) (Fig. 4A). The kinase activity of ROPs are related to virulence of parasites and modification of gene expression in host cells (Taylor et al., 2006; Saeij et al., 2007). This led us to explore whether the kinase activity of ROP39 was involved in host mitochondrial recruitment. To assess this aspect of ROP39, we generated a strain to express ROP39 without kinase activity in a *rop39*-deleted background (RH*Δrop39*:*ROP39δKAS*) by introducing ROP39 with a deleted kinase active site amino acids 402 to 414 (VHSDLKPENVLV) predicted in PROSITE (https://prosite. expasy.org/), into RH*Δrop39*. RH*Δrop39*:*ROP39δKAS* was not rescued in mitochondrial association (50.7±17.8%) (Fig. 4A), which indicates ROP39 recruits host mitochondria through its kinase activity. We confirmed that host mitochondria association declined in RH*Δrop39* (14.6±9.0%) in comparison with the RH strain (26.1±15.7%) in analysis of electron microscopy images (*P*<0.01, one-way ANOVA followed by Tukey–Kramer post hoc test) (Fig. 4C). On the other hand, host ER association was accelerated in RH*Δrop39* and the *maf1b*-deleted strain RHΔ*maf1b*, in order of RHΔ*maf1b* (39.6±16.3%) >RH*Δrop39* (27.1±11.4%) >RH (19.0±10.6%) (*P*<0.01, one-way ANOVA followed by Tukey–Kramer post hoc test) (Fig. 4C; Fig. S2). We wondered that the gene deletion lowered the fitness of parasites, resulting in decreased association of host mitochondria. To investigate whether *rop39*-deletion has any impact on the fitness of *T. gondii*, we performed a plaque assay. There were no significant differences in the mean plaque area and the number of plaques between RH parental strain and RH*Δrop39*, indicating that decreased association in RH*Δrop39* was not attributed to the gene disruption (Fig. S3). The percentage of PVs covered by host mitochondria or ER was almost constant (about 50%) in these three strains (Fig. 4D). This phenomenon happened possibly because ER associated the PVMs from which mitochondria were detached due to the disruption of ROP39 or MAF1b. Collectively, these findings show that ROP39 is a novel factor involved in recruitment of host mitochondria and the perimeter of PVs that can associate with host organelles may be fixed (Fig. 4E).

## DISCUSSION

**Fig. 5.**
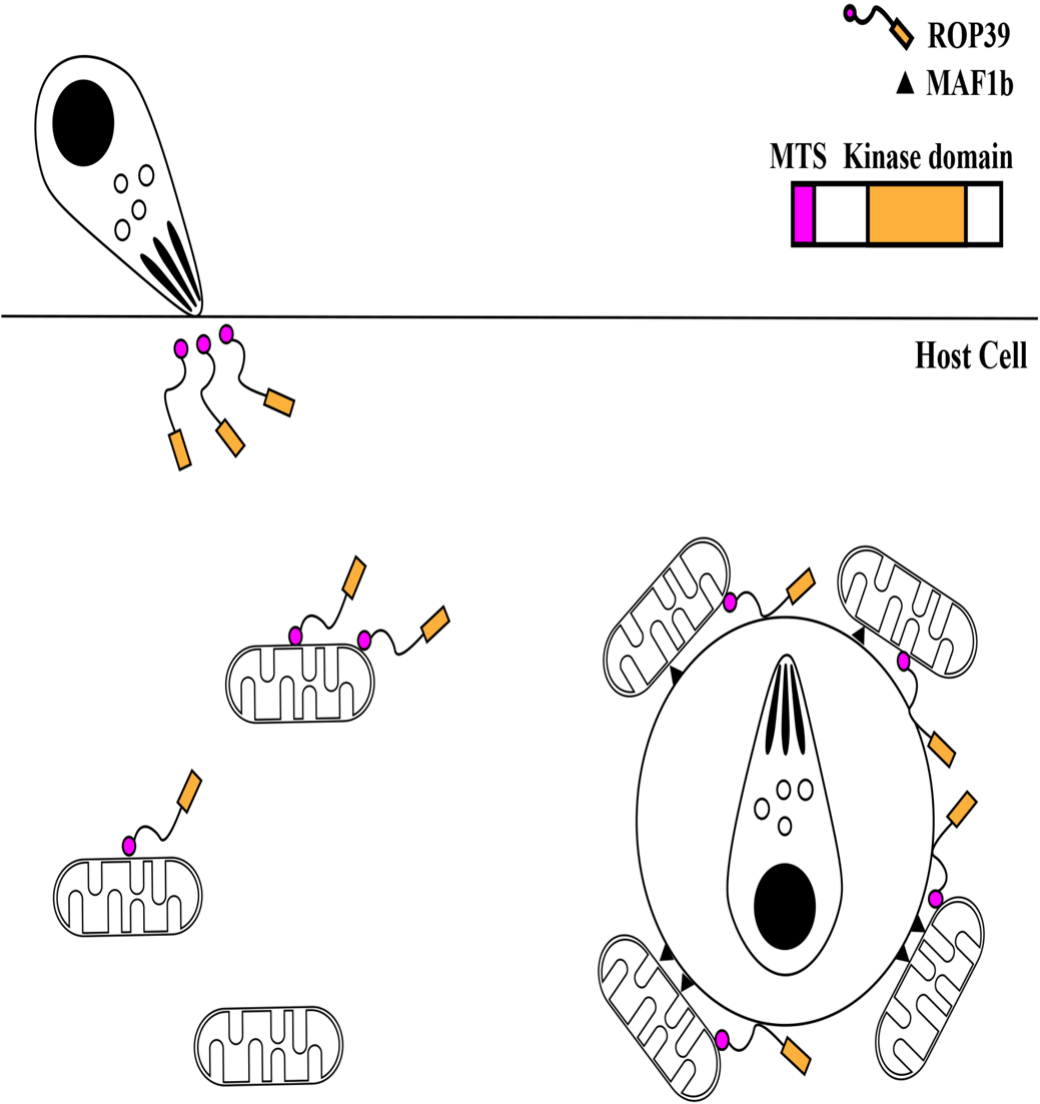
**The schematic image of our model related to host mitochondrial recruitment**.

Although MAF1b was identified as a mitochondrial association factor, the molecule(s) responsible for host mitochondrial recruitment was unraveled. Here, we showed that ROP39 seemed to be a causative molecule for the recruitment. We observed that mitochondrial association in *rop39*-knockout parasites was decreased compared with a parental strain, which supports that ROP39 works on host mitochondrial association. Considering the secretion timing of ROPs and localization of ROP39 in host cell, there is a high possibility that ROP39 binds to host mitochondria prior to PVMs formation. The unknown domain(s) of ROP39 or other parasite protein(s) may work on anchoring ROP39 to host mitochondria because proteins possessing an MTS are internalized when it is inserted into mitochondria. Moreover, substrate(s) of ROP39 may be related to the anchoring process because kinase-dead ROP39 could not rescue the level of host mitochondrial association in *rop39*-deleted mutants. Once the formation of PVMs occurs, ROP39 is recruited to PVMs possibly together with host mitochondria. Then, MAF1b may anchor host mitochondria on PVMs because it binds to host's mitochondrial interspace bridging (MIB) complex on its C-terminal region (Kelly et al., 2017), which can accelerate host mitochondrial association (Fig. 5).

The recruitment of host mitochondria was thought to be a means by which *T. gondii* acquires metabolites from host cells to help with its replication (Sinai et al., 1997; Crawford et al., 2006). However, it has been reported that host mitochondria associated with PV restrict the growth of *T. gondii* by intercepting the uptake of fatty acids (Pernas et al., 2018). However, it is unknown whether *T. gondii* exploit metabolites different from fatty acids through host mitochondrial recruitment. Further studies are needed to elucidate the reason that host mitochondrial association occurs in infected cells with *T. gondii*.

Collectively, our results identify *T. gondii* ROP39 as a new recruitment factor for host mitochondria. Exploiting components involved with recruitment of host mitochondria advances the understanding of why host mitochondrial recruitment diverges over a wide variety of intracellular parasites and how parasites employ mitochondrial recruitment to survive in host cells.

## MATERIALS AND METHODS

### Parasite and culture

RH (ATCC50838) strain was used in this study. The parasite was maintained in human foreskin fibroblasts (HFFs) cultured in Dulbecco's modified Eagle's medium (DMEM; Wako, Osaka, Japan) supplemented with 10% fetal bovine serum (FBS; Bovogen Biologicals, East Keilor, VIC, Australia), 2 mM L-glutamine (Sigma-Aldrich, St. Louis, MO, USA), 10 mM HEPES buffer (Sigma-Aldrich), and 10 µg/ml gentamicin (Sigma-Aldrich) (Nagamune et al., 2007), and was serially passaged at 37°C under 5% CO_2_.

### Mitochondria isolation and iTRAQ analysis

HFFs were cultivated to confluency in 145-mm dishes and 1.0×10^7^ parasites were inoculated and cultured at 37°C for 24 h. Host mitochondria were extracted from the HFF infected with *T. gondii* and the mixture of 1.0×10^7^ HFFs and 4.0×10^7^ *T. gondii* (as control) by using a Mitochondrial Isolation Kit for Cultured Cells (Thermo Fisher Scientific, Waltham, MA, USA), which was performed following the manufacturer's protocol. These samples were maintained at 4°C and shipped to Filgen, Inc. (Aichi, Japan). The proteins extracted from the samples were reduced, alkylated and digested, resulting in generation of peptides. Control peptides were labeled with 117 isobaric tags, and test peptides derived from host mitochondria in parasite-infected cells were labeled with 118 isobaric tags. The labeled peptides were separated using two-dimensional liquid chromatography followed by tandem mass spectrometry. Identification and quantification of proteins were performed using the Paragon™ algorithm in ProteinPilot™ software (AB Sciex, Framingham, MA, USA). The data from one technical replicate was processed in the analysis.

### Mammalian cell transfection

HFFs were cultivated to confluency on a cover slip in 12-well plates. The cells were transfected with mammalian transient vectors (pCMV-puro-P2A-BAFF, a kind gift from Dr Taishi Onodera, Department of Immunology, National Institute of Infectious Diseases, Japan) containing each *rop39* version using Lipofectamine 3000 (ThermoFisher Scientific).

pCMV empty vector was created from pCMV-puro-P2A-BAFF template using a PrimeSTAR Mutagenesis Basal Kit (Takara Bio Inc.) and the following primers: (forward primer: ATTCCACCGTGCGACGGCCGGCAAGC; reverse primer: GTCGCACGGTGGAA TTCAATCGATA). To construct pCMV-ROP39δSP-FLAG (ROP39δSP), SP-deleted *rop39* was amplified from genome DNA of *T. gondii* using a KOD FX Neo (Toyobo Co., Ltd., Osaka, Japan) and the following primers: (forward primer: ACACACGGTTGTATGTGGGT ACCTAGAACATTGCCAGT; reverse primer: ATGGTCTTTGTAATCAACAATTGATTCC CGAAGAGGC). PCR-amplified SP-deleted *rop39* was cloned into pCMV using a GeneArt Seamless PLUS Cloning and Assembly Kit (ThermoFisher Scientific) and the following primers: (forward primer: CGATTGAATTCCACCATGTGGGTACCTAGAACATTGCCAA CCATGTGGGTACCTAGAACATT; reverse primer: TGCCGGCCGTCGCACTTACTTGTC ATCGTCATCCTTGTAG). pCMV-ROP39δSPδMTS-FLAG (ROP39δSPδMTS) was created from ROP39δSP template using a PrimeSTAR Mutagenesis Basal Kit (Takara Bio Inc.) and the following primers: (forward primer: CCACCATGGATGTTGCACCTCATCAC; reverse primer: CAACATCCATGGTGGAATTCAATCG).

### Prediction of domain structure

Predictions for the signal peptide (SP), mitochondria targeting signal (MTS), kinase domain, and kinase active site were made using the following software: SignalP 3.0 (Bendtsen et al., 2004), Mitoprot (Fukasawa et al., 2015), SMART (Letunic et al., 2015; Letunic and Bork, 2018), and PROSITE (Sigrist et al., 2013).

### Generation of *rop39* and *maf1b* deletion mutants

To knockout the gene of interest (GOI), CRISPR/Cas9 system was employed. Briefly, single guide RNAs bind to the homologous region of GOI and CRISPR/Cas9 breaks the double-strand DNA on GOI. The wound DNA strands were repaired with a homologous recombination and GOI is replaced with a drug resistant gene flanked by the homologous regions (Jinek et al., 2012; Cong et al., 2013).

To create CRISPR/Cas9 expression vectors targeting GOI, single guide RNAs were introduced into pSAG1::CAS9-U6::sgUPRT (Addgene, https://www. addgene.org/) using a Q5 Site-Directed Mutagenesis Kit (New England Biolabs, Ipswich, MA, USA) to replace sgUPRT.

To create sgRNAs, forward primers for *rop39* AGGGCACTGGCTACAGTCGCGTTTTA GAGCTAGAAATAGC and for *maf1b* GCACGACAGTGAGATTCTTTGTTTTAGAGCT AGAAATAGC were designed using the E-CRISP program (Heigwer et al., 2014). The previously designed reverse primer for both genes AACTTGACATCCCCATTTAC was used (Shen et al., 2014).

To generate vectors to replace the GOI in homologous recombination, approximately 1-kb DNA fragments flanking the GOI were PCR-amplified using the following primers: *rop39* 5'-flanking region (forward primer: GGCCCCTTGTCAAATGACT; reverse primer: CGTCT TGACTGACAATGAACGGAT), *rop39* 3'-flanking region (forward primer: TTCTGAGGC TGTCGTCATTT; reverse primer: CTCTGCAACTGGCTTCACATC), *maf1b* 5'-flanking region (forward primer: ACATCCCACCAGACAGGAATTT; reverse primer: CTTTCCGTT CAGGGTGCCAT) and *maf1b* 3'-flanking region (forward primer: AGCGCGAGGAATCAG TTGT; reverse primer: TTCTGAAAAGCCTCCATCTACATTG). The pyrimethamine-resistant dihydrofolate reductase cassette (DHFR*) was flanked by PCR-amplified 5′- and 3′-flanking regions and cloned into a pUC19 vector (Takara Bio Inc., Shiga, Japan) at a restriction site of *Eco*RI.

The vectors for CRISPR/Cas9 expression and homologous recombination described above were electroporated into parasites using BTX Electro Cell Manipulator 600 (BTX, San Diego, CA, USA) (setting: 2.0 kV, 24 Ω). Transfectants were selected under 3 μM pyrimethamine (Wako) over 1 week and were cloned by limited dilution. To check for complete deletion of GOI, we performed PCR to amplify each DNA segment using the following primers: *rop39* 1f/1r (forward primer: TTCCCAAGAATCATCGTTTTTG; reverse primer: GCTTGATGATTTGTGAGGACGA), *rop39* 2f/2r (forward primer: AGAGTGCTGG ACTGTTGCTGTCT; reverse primer: TCTTGCTACTGAACAACATAAGGTTAG), *rop39* 3f/3r (forward primer: ATCTAAAACCGGAAAATGTGCTAGT; reverse primer: GTATAAT CGTGTTGGATGTGTCTGA), *rop39* 4f/4r (forward primer: AAGGATTCAGTCAACGGC A; reverse primer: AAGGTTAGGAGCGCTAGTTCAT), *maf1b* 1f/1r (forward primer: AAAT GGAGTGCTCCCAGATGAA; reverse primer: AGTAGAAAGGAATTAGCATAACTCAG T), *maf1b* 2f/2r (forward primer: TATCCACTCGTGAATGCGTTATC; reverse primer: TTC AGCCGACACA AACGG), *maf1b* 3f/3r (forward primer: TAGCTCAAGTCGAAGTCTGTT TTAC; reverse primer: CATGTACTGTAATTCGAAATTGGGG) (Fig. 2; Fig. S1).

### Generation of the stably transfected parasites

RH*Δrop39:ROP39* complemented strain and RH*Δrop39:ROP39δKAS* kinase-dead strain were generated from RH*Δrop39* strain to be transfected with pROP39-ROP39-FLAG or pROP39-ROP39δKAS-FLAG vectors, respectively. A chloramphenicol acetyltransferase (chloramphenicol resistance gene) contained plasmid was co-transfected together with each vector. The transfectants were cultured under 20 mM chloramphenicol for 2 weeks and cloned by limiting dilution.

To generate pROP39-ROP39-FLAG, approximately 1-kb DNA fragments flanking the coding region of *rop39* was PCR-amplified using the following primers: *rop39* 5'-flanking region (forward primer: GGCCCCTTGTCAAATGACT; reverse primer: CGTCTTGACTGAC AATGAACGGAT), *rop39* 3'-flanking region (forward primer: TTCTGAGGCTGTCGTCAT TT; reverse primer: CTCTGCAACTGGCTTCACATC). The coding region was flanked by PCR-amplified 5′- and 3′-flanking regions and cloned into a pUC19 vector (Takara Bio Inc.) at a restriction site of *Eco*RI.

To generate pROP39-ROP39δKAS-FLAG, the sequence of kinase active site (1204-GTTGTGCACTCGGATCTAAAACCGGAAAATGTGCTAGTC-1242) was deleted from pROP39-ROP39-FLAG by using a PrimeSTAR Mutagenesis Basal Kit (Takara Bio Inc.) and the following primers: (forward primer: GCTTGGGGACGAAGCCGGAAATTTG; reverse primer: GCTTCGTCCCCAAGCGCCTGAAGATG).

### Immunofluorescence assay

HFFs were cultivated to confluency on a cover slip (Matsunami Glass, Osaka, Japan) in 12-well plates and 1.0×10^6^ parasites were inoculated and cultured at 37°C for 24 h. The culture was treated with 3μM MitoTracker™ Red CMXRos (Thermo Fisher Scientific) and incubated at 37°C under 5% CO_2_ for 30 min. After washing with 1 mM D-PBS (Wako) with 100 µM CaCl_2_ (Wako), the culture was fixed with 4% paraformaldehyde (Polysciences Inc., Warrington, PA, USA) for 20 min. The fixed sample was washed with 1 mM D-PBS and permeabilized with 0.1% Triton X-100 (Sigma-Aldrich) for 20 min. After blocking with 10% FBS and 10% normal goat serum (NGS, Sigma-Aldrich), the sample was treated with mouse anti-FLAG or mouse anti-FLAG and rabbit anti-myc antibody (Santa Cruz Biotechnology, TX, USA) at a 1:1000 dilution for 1.0 h and then with Alexa Fluor 488-conjugated goat anti-mouse (Life Technologies, Carlsbad, CA, USA) or Alexa Fluor 488-conjugated goat anti-mouse and Alexa Fluor 568-conjugated goat anti-rabbit secondary antibody (Life Technologies) at a 1:1000 dilution for 30 min followed by observation using a laser scanning microscope (LSM780, Carl Zeiss, Oberkochen, Germany). Parasites transiently transfected with pSAG1-ROP39-FLAG or pSAG1-ROP39-FLAG and pSAG1-MAF1b-myc were used in this assay. *rop39* and *maf1b* were amplified and cloned into the restriction site downstream of SAG1 promoter (TGME49_233460) by using the following primers: *rop39* (forward primer: TAAACACACGGTTGTATGAGCAAACCTTTTTTCCCAC; reverse primer: ATGGTCTTTGTAATCAACAATTGATTCCCGAAGAGGC), *maf1b* (forward primer: TAAACACACGGTTGTATGTGGCGCATCTGGAGAT; reverse primer: AATCAACTTTT GTTCGTCCAGCATGCTAGCCAGATA).

### Transmission electron microscopy

HFFs were cultivated to confluency on a gold grid in 12-well plates and 1.0×10^6^ parasites were inoculated and cultured at 37°C for 24 h. The culture was freeze-dried in liquid nitrogen and chemical fixation was performed. The sample was scanned on a transmission electron microscope (Tokai-Denshi Inc., Aichi, Japan). Host mitochondria and ER were distinguished by its membranous structure and electron density.

### Quantification of the strength of host organelle recruitment

The perimeter of PVs and the length of host mitochondria and ER in contact with PVs were measured using ImageJ software (http://imagej.nih.gov/ij/) in immunofluorescence and electron microscopy images. A total length of mitochondria and/or ER in contact with PVs was divided by the perimeter of PVs and the ratio was defined as the strength of host organelle recruitment.

### Evacuole assay

Parasites were collected by scraping the cell monolayer and released from host cells by passage through a 21-gauge needle. Extracellular parasites were filtered onto a polycarbonate membrane filter (3.0-μm pore size; Millipore, Bedford, MA, USA) and washed with Hanks' balanced salt solution (Wako) containing 0.1 mM EGTA and 10 mM HEPES. HFFs were cultivated on a cover slip (Matsunami Glass, Osaka, Japan) in 12-well plates for 48 h before the experiment. Parasites were pretreated with 1 μM cytochalasin D (Sigma-Aldrich) at room temperature for 10 min. 1.0×10^8^ parasites were inoculated on host cells and cultured at 37°C for 60 min in the presence of 1 μM cytochalasin D. After washing with 1 mM D-PBS, the culture was fixed with 4% paraformaldehyde at 4°C for 20 min.

### Plaque assays

HFFs were cultivated to confluency in six-well plates and 250 parasites were inoculated and cultured at 37°C for 11 days without disturbance. After washing with 1 mM D-PBS, the culture was fixed with methanol (Sigma-Aldrich) at 4°C for 20 min. The fixed culture was treated with 0.25% CBB R-250 (Wako) for 3 h. The images of plaques were procced on ImageJ software (http://imagej.nih.gov/ij/) by calculating the area size of all plaques. The plaque area was defined manually and marked with lines to major the area size by using Tools in Polygon Section.

### Statistical analysis

All data are shown as means or mean±s.d. The normality was checked by Shapiro-Wilk test. All statistical tests were performed using R software (https://www.r-project.org).

## References

[BIO058988C1] Bendtsen, J. D., Nielsen, H., von Heijne, G. and Brunak, S. (2004). Improved prediction of signal peptides: SignalP 3.0. *J. Mol. Biol.* 340, 783-795. 10.1016/j.jmb.2004.05.028 15223320

[BIO058988C2] Boothroyd, J. C. and Dubremetz, J. F. (2008). Kiss and spit: the dual roles of *Toxoplasma* rhoptries. *Nat. Rev. Microbiol.* 6, 79-88. 10.1038/nrmicro1800 18059289

[BIO058988C3] Cong, L., Ran, F. A., Cox, D., Lin, S., Barretto, R., Habib, N., Hsu, P. D., Wu, X., Jiang, W., Marraffini, L. A. et al. (2013). Multiplex genome engineering using CRISPR/Cas systems. *Science* 339, 819-824. 10.1126/science.1231143 23287718 PMC3795411

[BIO058988C4] Crawford, M. J., Thomsen-Zieger, N., Ray, M., Schachtner, J., Roos, D. S. and Seeber, F. (2006). *Toxoplasma gondii* scavenges host-derived lipoic acid despite its de novo synthesis in the apicoplast. *EMBO J.* 25, 3214-3222. 10.1038/sj.emboj.7601189 16778769 PMC1500979

[BIO058988C5] Fukasawa, Y., Tsuji, J., Fu, S.-C., Tomii, K., Horton, P. and Imai, K. (2015). MitoFates: Improved prediction of mitochondrial targeting sequences and their cleavage sites. *Mol. Cell. Proteomics.* 14, 1113-1126. 10.1074/mcp.M114.043083 25670805 PMC4390256

[BIO058988C6] Grigg, M. E. and Sundar, N. (2009). Sexual recombination punctuated by outbreaks and clonal expansions predicts Toxoplasma gondii population genetics. *Int. J. Parasitol.* 39, 925-933. 10.1016/j.ijpara.2009.02.005 19217909 PMC2713429

[BIO058988C7] Heigwer, F., Kerr, G. and Boutros, M. (2014). E-CRISP: fast CRISPR target site identification. *Nat. Methods.* 11, 122-123. 10.1038/nmeth.2812 24481216

[BIO058988C8] Horwitz, M. A., John, A., Hartford, G. L. and Fellowship, F. (1983). Formation of a novel phagosome by the Legionnaires’ disease bacterium (*Legionella pneumophila*) in the human monocytes. *J. Exp. Med.* 158, 1319-1331. 10.1084/jem.158.4.1319 6619736 PMC2187375

[BIO058988C9] Jinek, M., Chylinski, K., Fonfara, I., Hauer, M., Doudna, J. A. and Charpentier, E. (2012). A programmable dual-RNA-guided DNA endonuclease in adaptive bacterial immunity. *Science* 337, 816-822. 10.1126/science.1225829 22745249 PMC6286148

[BIO058988C10] Kelly, F. D., Wei, B. M., Cygan, A. M., Parker, M. L., Boulanger, M. J. and Boothroyd, J. C. (2017). *Toxoplasma gondii* MAF1b binds the host cell MIB complex to mediate mitochondrial association. *mSphere* 2, 1-14. 10.1128/mSphere.00183-17 PMC5444011 28567444

[BIO058988C11] Letunic, I. and Bork, P. (2018). 20 years of the SMART protein domain annotation resource. *Nucleic Acids Res.* 46, 493-496. 10.1093/nar/gkx922 29040681 PMC5753352

[BIO058988C12] Letunic, I., Doerks, T. and Bork, P. (2015). SMART: recent updates, new developments and status in 2015. *Nucleic Acids Res.* 43, 257-260. 10.1093/nar/gku949 PMC4384020 25300481

[BIO058988C13] Matsumoto, A., Bessho, H., Uehira, K. and Suda, T. (1991). Morphological studies of the association of mitochondria with chlamydial inclusions and the fusion of chlamydial inclusions. *J. Electron Microsc.* 40, 356-363. 1666645

[BIO058988C14] Montoya, J. G. and Liesenfeld, O. (2004). Toxoplasmosis. *Lancet* 363, 1965-1976. 10.1016/S0140-6736(04)16412-X 15194258

[BIO058988C15] Nagamune, K., Beatty, W. L. and Sibley, L. D. (2007). Artemisinin induces calcium-dependent protein secretion in the protozoan parasite *Toxoplasma gondii*. *Eukaryot. Cell.* 6, 2147-2156. 10.1128/EC.00262-07 17766463 PMC2168421

[BIO058988C16] Pernas, L. and Boothroyd, J. C. (2010). Association of host mitochondria with the parasitophorous vacuole during *Toxoplasma* infection is not dependent on rhoptry proteins ROP2/8. *Int. J. Parasitol.* 40, 1367-1371. 10.1016/j.ijpara.2010.07.002 20637758 PMC2939271

[BIO058988C17] Pernas, L., Adomako-Ankomah, Y., Shastri, A. J., Ewald, S. E., Treeck, M., Boyle, J. P. and Boothroyd, J. C. (2014). *Toxoplasma* effector MAF1 mediates recruitment of host mitochondria and impacts the host response. *PLoS Biol.* 12, e1001845. 10.1371/journal.pbio.1001845 24781109 PMC4004538

[BIO058988C18] Pernas, L., Bean, C., Boothroyd, J. C. and Scorrano, L. (2018). Mitochondria restrict growth of the intracellular parasite *Toxoplasma gondii* by limiting its uptake of fatty acids. *Cell Metab.* 27, 886-897. 10.1016/j.cmet.2018.02.018 29617646

[BIO058988C19] Saeij, J. P. J., Coller, S., Boyle, J. P., Jerome, M. E., White,M. W. and Boothroyd, J. C. (2007). *Toxoplasma* co-opts host gene expression by injection of a polymorphic kinase homologue. *Nature* 445, 324-327. 10.1038/nature05395 17183270 PMC2637441

[BIO058988C20] Scanlon, M., Leitch, G. J., Visvesvara, G. S. and Shaw, A. P. (2004). Relationship between the host cell mitochondria and the parasitophorous vacuole in cells infected with Encephalitozoon Microsporidia. *J. Eukaryot. Microbiol.* 51, 81-87. 10.1111/j.1550-7408.2004.tb00166.x 15068269

[BIO058988C21] Shen, B., Brown, K. M., Lee, T. D. and Sibley, L. D. (2014). Efficient gene disruption in diverse strains of *Toxoplasma gondii* using CRISPR/CAS9. *MBio* 5, 1-11. 10.1128/mBio.01114-14 PMC4030483 24825012

[BIO058988C23] Sigrist, C. J., de Castro, E., Cerutti, L., Cuche, B. A., Hulo, N., Bridge, A., Bougueleret, L. and Xenarios, I. (2013). New and continuing developments at PROSITE. *Nucleic Acids Res.* 41, 344-347. 10.1093/nar/gks1067 PMC3531220 23161676

[BIO058988C24] Sinai, A. P. and Joiner, K. A. (2001). The Toxoplasma gondii protein ROP2 mediates host organelle association with the parasitophorous vacuole membrane. *J. Cell Biol.* 154, 95-108. 10.1083/jcb.200101073 11448993 PMC2196872

[BIO058988C25] Sinai, A. P., Webster, P. and Joiner, K. A. (1997). Association of host cell endoplasmic reticulum and mitochondria with the *Toxoplasma gondii* parasitophorous vacuole membrane: a high affinity interaction. *J. Cell. Sci.* 110, 2117-2128. 10.1242/jcs.110.17.2117 9378762

[BIO058988C26] Tahara, M., Andrabi, S. B. A., Matsubara, R., Aonuma, H. and Nagamune, K. (2016). A host cell membrane microdomain is a critical factor for organelle discharge by *Toxoplasma gondii*. *Parasitol. Int.* 65, 378-388. 10.1016/j.parint.2016.05.012 27217289

[BIO058988C27] Taylor, S., Barragan, A., Su, C., Fux, B., Fentress, S. J., Tang, K., Beatty, W. L., Hajj, H. E., Jerome, M., Behnke, M. S. et al. (2006). A secreted serine-threonine kinase determines virulence in the eukaryotic pathogen *Toxoplasma gondii*. *Science* 314, 1776-1780. 10.1126/science.1133643 17170305

[BIO058988C28] Wiese, S., Reidegeld, K. A., Meyer, H. E. and Warscheid, B. (2007). Protein labeling by iTRAQ: A new tool for quantitative mass spectrometry in proteome research. *Proteomics* 7, 340-350. 10.1002/pmic.200600422 17177251

